# Second-line treatment after sunitinib therapy in patients with renal cell carcinoma: a comparison of axitinib and mammalian target of rapamycin inhibitors

**DOI:** 10.18632/oncotarget.26439

**Published:** 2018-12-11

**Authors:** Satoshi Tamada, Taro Iguchi, Minoru Kato, Sayaka Yasuda, Takeshi Yamasaki, Tatsuya Nakatani

**Affiliations:** ^1^ Department of Urology, Osaka City University Graduate School of Medicine, Abeno-ku, Osaka 545–8585, Japan

**Keywords:** molecular targeted therapy, renal cell carcinoma, second-line treatment, axitinib, mammalian target of rapamycin

## Abstract

This retrospective study compared the outcomes of sequential therapy using sunitinib followed by axitinib or the mammalian target of rapamycin (mTOR) inhibitors (everolimus or temsirolimus). Among 234 patients treated with molecular-targeted drugs for metastatic renal cell carcinoma, we selected 137 patients treated with sunitinib as the first-line therapy. We then compared patients treated with axitinib (n = 52) or mTOR inhibitors (n = 31), as the second-line treatment, and investigated the progression-free survival (PFS) and overall survival (OS). The PFS of axitinib-treated patients (median 8.7 months) was superior to that of mTOR inhibitors-treated patients (median 3.4 months; P = 0.001). Additionally, the OS from baseline of axitinib-treated patients (median 69 months) was superior to that of mTOR inhibitors-treated patients (median 33.4 months; P = 0.034). A multivariate analysis was performed with the following factors: the drugs used for the second-line treatment, the Memorial Sloan Kettering Cancer Center risk classification during the initial treatment, whether the discontinuation of the first-line treatment was due to adverse events, and whether the duration of response of the first-line treatment was less than 6 or 12 months. Importantly, the drugs used for the second-line treatment and Memorial Sloan Kettering Cancer Center risk classification were independent factors. Our findings suggest that axitinib works better than mTOR inhibitors after the first-line treatment with sunitinib.

## INTRODUCTION

The replacement of cytokines with molecular-targeted drugs has achieved prolongation of overall survival (OS) in the treatment of metastatic renal cell carcinoma (mRCC) [1, 2]. An increase in the molecular-targeted drug alternatives is the underlying reason for the prolongation of OS [3–8]. Interestingly, sequential therapy using these drugs has been studied and has been reported that prognosis was better when sunitinib was switched to sorafenib, rather than to temsirolimus. In other words, tyrosine kinase inhibitor (TKI)-TKI sequence therapy is superior to TKI-mammalian target of rapamycin (mTOR) inhibitor therapy [9]. Recently, in Japan, it is a common practice to use sunitinib as the first-line treatment, followed by axitinib [10]. This is supported by the AXIS trial that demonstrated the contribution of axitinib to the prolongation of progression-free survival (PFS) was higher than that of sorafenib as the second-line treatment following sunitinib [11]. Additionally, a study in Japan revealed that the median OS was 27 months in patients treated with axitinib as the second-line treatment [12]. However, it should be noted that these studies reported outcomes from the second-line treatment and do not indicate the OS after the initiation of sunitinib. To elucidate the importance of sequential therapy, it is important to calculate the OS from baseline treatment. Therefore, we conducted this study to compare the outcomes of the sequential therapy that used sunitinib followed by either axitinib or mTOR inhibitors and clarify the outcomes of sequential therapy in the real-world setting.

## RESULTS

### Patient characteristics

Table 1 shows the characteristics of the study population. Among 234 patients treated with molecular-targeted drugs for mRCC, 137 patients were treated with sunitinib as the first-line therapy.

**Table 1:** Characteristics of the study population and treatments (n = 234)

Age (years) (median)		67	range: 35–84
Sex	Male	180	
	Female	54	
The Memorial Sloan Kettering Cancer Center risk classification	Favorable (%)	50	(21.4)
	Intermediate (%)	126	(53.8)
	Poor (%)	49	(20.9)
	unknown (%)	9	(3.8)
Sites of metastasis	Lung	153	
	Lymph node	59	
	Bone	67	
	Pancreas	14	
	Liver	20	
	Brain	13	
Prior nephrectomy	Yes (%)	219	(93.6)
	No (%)	15	(6.4)
Molecular targeted drugs			
1st-line	Sunitinib	137	
	Sorafenib	75	
	Pazopanib	10	
	Temsilorimus	12	
2nd-line	mTORi	43	
	Axitinib	57	
	Sunitinib	25	
	Pazopanib	2	
	Nivolumab	5	
	Sorafenib	5	

Abbreviations: mTORi; mammalian target of rapamycin inhibitors.

### Treatment effects of sunitinib and sorafenib

Time to treatment failure (TTF) and OS of the patients treated with sunitinib (50 mg was administered orally every day for over 2 or 4 weeks, followed by a 1- or 2-week washout period) and sorafenib (400 mg was administered orally twice a day continuously) are shown in Supplementary Figure 1. The patients treated with sunitinib (median 69.5 months) had a significantly prolonged survival compared with those treated with sorafenib (median 33.5 months; p = 0.0488) (Supplementary Figure 1a). Interestingly, the TTF with sorafenib (median 12.8 months) was superior to sunitinib (median 7.4 months; p = 0.020) (Supplementary Figure 1b). Among the patients treated with sunitinib as the first-line treatment (excluding consecutive patients), 28 patients (20.4%) completed the treatment with sunitinib alone. Additionally, 41 patients (54.7%) treated with sorafenib as the first-line treatment required no further treatment.

### Effects of second-line treatment with axitinib or mTOR inhibitors

**Figure 1:**
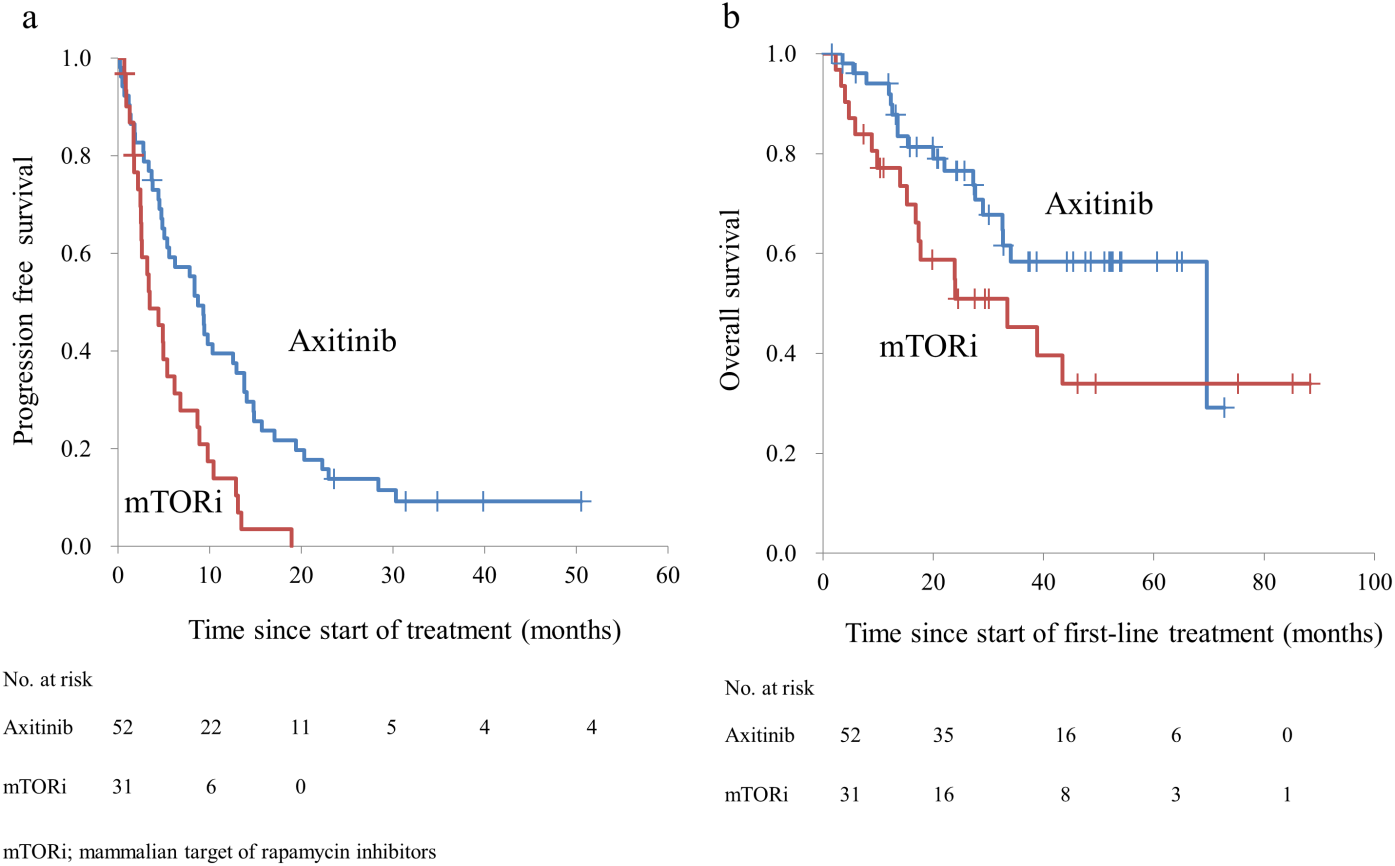
**(a)** Progression-free survival of patients with metastatic renal cell carcinoma after receiving axitinib and mammalian target of rapamycin inhibitors. **(b)** Overall survival of patients with metastatic renal cell carcinoma after receiving sunitinib followed by the second-line treatments.

Table 2 shows the characteristics of the patients treated with sunitinib, followed by either axitinib (10 mg per day administered orally, with allowed dose escalation of up to 20 mg) or mTOR inhibitors (everolimus, 10 mg per day administered orally or temsirolimus, 25 mg per week administered via intravenous drip) as the second-line treatment. The treatment with axitinib significantly prolonged the PFS (median 8.7 months) compared with that by mTOR inhibitors (median 3.4 months; p = 0.001) (Figure 1a). Similar effects were observed with respect to OS after the initiation of sunitinib when treatment with axitinib (median 69.5 months) was compared with mTOR inhibitors (median 33.4 months; p = 0.034) (Figure 1b).

**Table 2:** Characteristics of patients treated with sunitinib followed by the second-line treatments (n = 83)

		Axitinib (n = 52)		mTORi (n = 31)		p value
Age (years) (median)		68	range: 41–84	63	range: 43–77	0.06
Sex	Male (%)	40	(76.9)	21	(67.7)	0.36
	Female (%)	12	(23.1)	10	(32.3)	
Number of metastatic organs	Single (%)	29	(55.8)	15	(48.4)	0.51
	Multiple (%)	23	(44.2)	16	(51.6)	
Sites of metastasis	Lung	37		23		0.57
	Lymph node	6		9		
	Bone	15		9		
	Pancreas	3		1		
	Liver	4		1		
	Brain	3		2		
The Memorial Sloan Kettering Cancer Center risk classification	Favorable (%)	11	(21.2)	10	(32.3)	0.50
	Intermediate (%)	29	(55.8)	14	(45.2)	
	Poor (%)	11	(21.2)	7	(22.6)	
	unknown (%)	1	(1.9)	0	0.0	
Reason for discontinuation of first-line drug	Progressive disease (%)	39	(75.0)	26	(83.9)	0.34
	Adverse event (%)	13	(25.0)	5	(16.1)	
Histology	Clear cell (%)	50	(96.2)	27	(87.1)	0.13
	Non-clear cell (%)	2	(3.8)	4	(12.9)	
Sunitinib response period	within 6 months (%)	26	(50.0)	15	(48.4)	0.89
	over 6 months (%)	26	(50.0)	16	(51.6)	
	within 12 months (%)	36	(69.2)	23	(74.2)	0.63
	over 12 months (%)	16	(30.8)	8	(25.8)	

Abbreviations: mTORi; mammalian target of rapamycin inhibitors.

### Univariate analysis

We examined the factors that influenced the OS of these patients (Table 3). The results of univariate analysis revealed that axitinib improved the survival rate more significantly than that by mTOR inhibitors (hazard ratio 0.47). When analyzed using the Memorial Sloan Kettering Cancer Center (MSKCC) risk classification, the survival rate was significantly superior in the favorable group (hazard ratio 0.07) and significantly inferior in the poor group than in the other groups (hazard ratio 3.61).

**Table 3:** Results of Cox proportional stepwise multivariate analysis for the association between the variables and overall survival

Comparison			Overall survival (months) (median)			Unadjusted		Adjusted	
						HR (95% CI)	p value	HR (95% CI)	p value
Axitinib	vs.	mTORi	69.5	vs.	33.5	0.47 (0.24–0.92)	0.028	0.29 (0.14–0.62)	0.001
Discontinuing first-line treatment for toxicity	vs.	Discontinuing first-line treatment for progression	not reached	vs.	20.1	0.44 (0.15–1.24)	0.120	0.84 (0.27–2.65)	0.770
MSKCC risk classification Favorable	vs.	MSKCC risk classification Others	not reached	vs.	19.3	0.07 (0.01–0.51)	0.009	0.08 (0.01–0.59)	0.014
MSKCC risk classification Poor	vs.	MSKCC risk classification Others	12.4	vs.	not reached	3.61 (1.80–7.25)	<0.001	3.04 (1.46–6.34)	0.003
Response duration of sunitinib < 6 months	vs.	Response duration of sunitinib ≥ 6 months	24.1	vs.	27.1	0.74 (0.38–1.45)	0.385	0.91 (0.45–1.86)	0.804

Abbreviations: mTORi; mammalian target of rapamycin inhibitors.

MSKCC; the Memorial Sloan Kettering Cancer Center risk classification.

HR; hazard ratio.

CI; confidence interval.

The analysis of whether the first-line treatment was discontinued due to AEs and whether the response duration to sunitinib was less or more than 6 months revealed no difference in the OS. A similar result was observed when we analyzed whether the response duration to sunitinib was less or more than 12 months (OS for response duration < 12 months; median 24.1 months, OS for response duration ≥ 12 months; median 21.3 months, p = 0.461).

### Multivariate analysis

Among the patients (n = 83) who received second-line treatment, 35 patients died. The results of multivariate analysis revealed that the treatment drugs (axitinib or mTOR inhibitors) and the MSKCC risk classification were independent prognostic factors.

### Sunitinib - axitinib sequence therapy

To clarify the relevance of sunitinib and axitinib, we further investigated sequential therapy with sunitinib, axitinib. In the patients treated with sunitinib followed by axitinib, no difference was observed when we analyzed whether the TTF of sunitinib was less or more than 6 months (median PFS of axitinib: 5.6 months [TTF of sunitinib for < 6 months] vs. 9.8 months [TTF of sunitinib for ≥ 6 months]; p = 0.562). Similar results was observed when we analyzed whether the TTF was less or more than 12 months (median PFS of axitinib: 6.2 months [TTF of sunitinib for < 12 months] vs. 9.8 months [TTF of sunitinib for ≥ 12 months], p = 0.946) and the OS (median OS from second-line treatment: not reached [TTF of sunitinib for < 6 months] vs. 24.8 months [TTF of sunitinib for ≥ 6 months], p = 0.835, not reached [TTF of sunitinib for < 12 months] vs. not reached [TTF of sunitinib for ≥ 12 months], p = 0.882).

### Adverse events

The adverse events (AEs) leading to treatment discontinuation included gastrointestinal perforation, renal dysfunction, perianal abscess, diarrhea, hyponatremia and hoarseness due to axitinib, dermatitis, stomatitis, and skin rash due to mTOR inhibitors.

## DISCUSSION

In this study, we found that in patients initially treated with sunitinib, axitinib had a superior OS than mTOR inhibitors when administered as the second-line treatment. In the RECORD-1 trial, everolimus significantly prolonged the OS compared with the placebo [6]. However, in the AXIS trial, axitinib significantly prolonged the PFS but failed to show a significant effect on the OS [7]. Everolimus and axitinib are not comparable because different comparators were used in these trials. Hutson et al. compared sorafenib and temsirolimus as the second-line drugs and found that the former was superior [9]. However, to the best of our knowledge, our study is the first that proved the superiority of axitinib as the second-line treatment in Asian population, although two retrospective studies have revealed that axitinib and everolimus as the second-line drugs have similar effect in Caucasians [13, 14].

To evaluate the effect of the second-line therapy agent on the survival rate, it is necessary to narrow down the first-line treatment drugs. At our institution, sorafenib was used as the first-line treatment until the introduction of molecular-targeted drugs in 2008 in Japan. In this study, we examined whether sorafenib or sunitinib was appropriate as the first-line treatment. The prolongation of TTF by sorafenib was better than that by sunitinib, whereas the prolongation of OS by sunitinib was better than that by sorafenib. As only these two drugs were available for the treatment of metastatic renal cancer in the past, the discrepancies in TTF and OS might be because most patients treated with sorafenib tended to complete their treatment with only one drug. Compared with that of the placebo, the treatment with sorafenib prolonged the PFS of patients with mRCC in whom previous therapy has failed [3]. Sorafenib, under the current guidelines, is not recommended as the first-line treatment drug [15]. In contrast, sunitinib, as the first-line treatment, demonstrated longer OS, and improved response and PFS of patients with mRCC than those with IFN-alfa [5, 16]. Therefore, we investigated the second-line treatment in patients administered sunitinib as the first-line treatment. Our results revealed that axitinib is a superior second-line treatment than mTOR inhibitors, but this interpretation is probably subject to various factors.

Patients who discontinued the use of the first-line treatment due to AEs had a favorable prognosis compared with that in patients who discontinued treatment due to disease progression [17]. However, the discontinuation of treatment due to AEs was not an independent prognostic factor in our study. A non-significant difference was observed in the univariate analysis, which might be due to the limited number of cases.

It has been reported that the response period of the first-line treatment affected the treatment effect of the second-line drugs. In the AXIS trial, patients who started first-line treatment with sunitinib with time to progression (TTP) ≥ 10 months had longer OS than those with TTP < 10 months with the second-line treatment with axitinib [11]. However, D'Aniello et al. reported no significant difference in the survival rate due to axitinib between patients who ended up with the treatment period of ≥ 13.2 months and those with < 13.2 months in the first-line treatment with sunitinib [18]. The cut-off value in these trials was defined as the median OS of each patient group. In this study, we examined the response period of the first-line treatment at both 6 and 12 months; however, neither was an independent prognostic factor. The reason to examine the response period at month 12 was that it was close to the mean response period of sunitinib. Additionally, we investigated at 6 months to evaluate whether the effects of axitinib were influenced in the early-stage non-response patients treated with sunitinib. Based on our findings, we concluded that there was no relationship between the response period of sunitinib and the effect of axitinib.

The MSKCC [19] and the International Metastatic Renal Cell Carcinoma Database Consortium risk classification [20] are widely used for the prognostic prediction of mRCC, and their efficacy has been reported [21, 22]. Our study focused on patients treated with sunitinib as the first-line drug and reconfirmed that the stratification of survival rate using the MSKCC risk classification was the independent prognostic factor. We hypothesized that it is necessary to take into consideration the risk classification at baseline to select an appropriate drug when proceeding to the second-line treatment. Patients who are classified into the favorable group or the intermediate group, based on the risk classification at baseline, should select a drug that has a high anti-tumor effect as they have longer survival than those classified into the poor group. It has also been reported that with both the first-line [23] and second-line treatments [24, 25], the OS was longer in patients treated with a drug with high cytoreductive effect. Miyake et al. reported that axitinib has a superior cytoreductive effect when administered as a second-line treatment drug [12]. Taken together, axitinib is thought to be a superior second-line treatment drug than the mTOR inhibitors.

This was a retrospective study and had certain limitations. We used Cox proportional stepwise multivariate analysis to evaluate the association between several factors and OS. However, the sample size was too small to use this analysis and this was a retrospective study. Everolimus and temsirolimus were used for patients receiving mTOR inhibitors as the second-line treatment. Iacovelli et al. reported a significant difference between these drugs as the second-line treatment [26]. Patients treated with second-line molecular-targeted drugs were selected according to the judgment of the attending physician. Notably, treatment selection bias may be present.

During recent years, it has been reported that nivolumab significantly prolonged the OS compared with that by mTOR inhibitors [27], and it has been recommended as a second-line drug. However, in the absence of studies comparing axitinib with nivolumab, a suitable second-line treatment remains unclear. Although the number of cases in which nivolumab is used as a second-line drug is increasing, it is limited and the follow-up period is short. Further studies are required to investigate potential second-line treatment drugs.

In summary, sequential therapy using sunitinib and axitinib significantly prolonged the OS compared with that by sunitinib and a mTOR inhibitor.

## MATERIALS AND METHODS

### Study design

Since 2008, 234 patients received molecular-targeted drugs for the treatment of mRCC at the Osaka City University. From this group, we retrospectively analyzed patients (n = 212) administered sunitinib or sorafenib as the first-line treatment. Further, we examined the differences between the TTF and OS in patients who were treated with each drug. Further, in patients treated with sunitinib as the first-line treatment (n = 137), we analyzed the differences in the PFS and OS of patients treated with either axitinib (n = 52) or mTOR inhibitors (everolimus, n = 20 or temsirolimus, n = 11) (n = 31) as the second-line treatment. Patients treated with the second-line molecular-targeted drugs were selected according to the judgment of the attending physician due to the AEs of sunitinib or other complications. Permission to access the database to review the medical records of patients was obtained from the Local Research Ethics Committee at Osaka City University (approval number 3441).

### Univariate and multivariate analyses

The OS was classified based on the following four factors to perform the univariate and multivariate analyses: the type of second-line treatment, MSKCC risk classification at initial treatment, whether the first-line drugs were discontinued due to AEs, and whether the response duration of sunitinib was less or more than 6 or 12 months, respectively. The factor 'response duration of sunitinib' was analyzed at either 6 or 12 months.

### Sunitinib-axitinib sequential therapy

We analyzed the relationship between the response duration of sunitinib and the effect of axitinib in patients who received sunitinib followed by axitinib to clarify the relationship between the response period of sunitinib and effect of axitinib.

### Follow-up schedule and outcome measurement

Response assessment was performed by computed tomography or magnetic resonance imaging scans every 10–12 weeks and evaluated according to the Response Evaluation Criteria in Solid Tumors (RECIST) v.1.1.[28].

### Statistical analyses

All statistical analyses were performed using Microsoft Excel^®^ (Microsoft, Redmond, Washington, USA). Differences in clinicopathological variables between axitinib and mTOR inhibitors were analyzed by the chi-squared analysis. The OS was estimated by Kaplan–Meier method, and the differences were determined using the log-rank test. Statistical significance was set at p-value <0.05.

## SUPPLEMENTARY MATERIALS FIGURE



## Abbreviations

OS: overall survival
mRCC: metastatic renal cell carcinoma
PFS: progression-free survival
TTF: time to treatment failure
MSKCC: Memorial Sloan Kettering Cancer Center
AEs: adverse events
TTP: time to progression
