# Supplementary material for: Second-line treatment after sunitinib therapy in patients with renal cell carcinoma: a comparison of axitinib and mammalian target of rapamycin inhibitors
Source: Oncotarget. 2018 Dec 11;9(97):37017–25. doi: 10.18632/oncotarget.26439 (PMC6319347; doi:10.18632/oncotarget.26439)
Supplement: Supplementary file 1 [file oncotarget-09-37017-s001.pdf]

Second-line treatment after sunitinib therapy in patients with renal cell carcinoma: a comparison of axitinib and mammalian target of rapamycin inhibitors

SUPPLEMENTARY MATERIALS

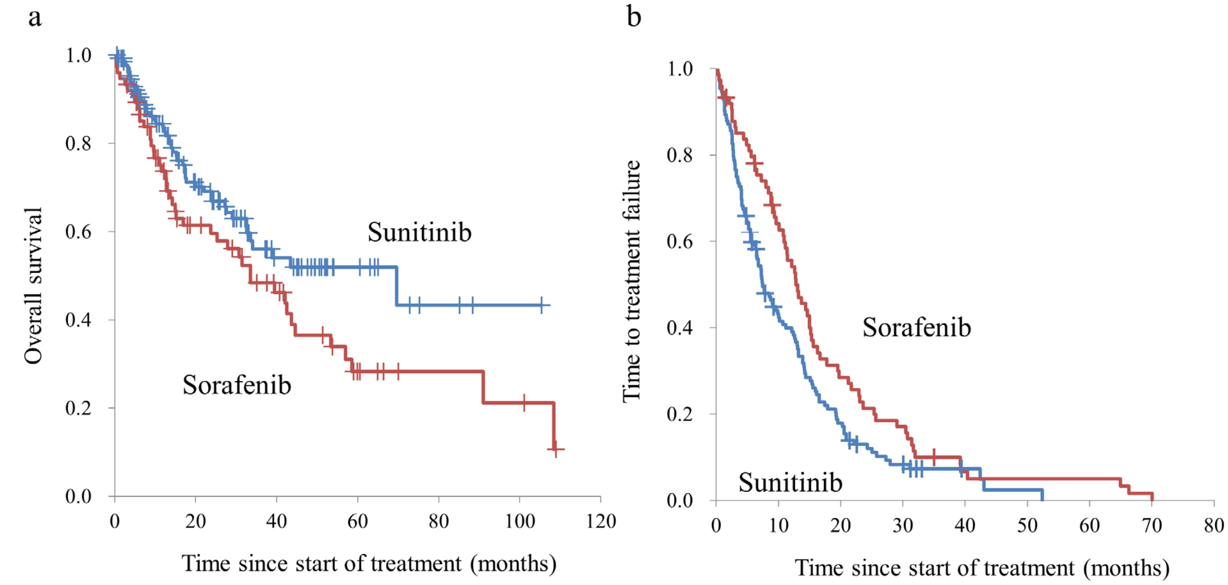

No. at risk

Sunitinib	137	70	27	11	4	3
Sorafenib	75	37	22	9	5	5

No. at risk

Sunitinib	137	52	22	9	4	2	2
Sorafenib	75	45	21	13	5	3	0

Supplementary Figure 1: Overall survival **(a)** and time to treatment failure **(b)** of patients with metastatic renal cell carcinoma after receiving sunitinib or sorafenib as the first-line treatment.
